# Screening for Cervical Cancer in Pregnancy

**DOI:** 10.3389/or.2023.11429

**Published:** 2023-10-17

**Authors:** Sarita Kumari

**Affiliations:** All India Institute of Medical Sciences, New Delhi, India

**Keywords:** cervical cancer, screening, pregnancy, prevention, HPV DNA

## Abstract

Cervical cancer remains a leading cause of cancer related morbidity and mortality in low/low-middle income countries. Lack of screening is the leading cause of cases being diagnosed in advanced stages and screening is still opportunistic in a majority of these countries. Hospital visits during pregnancy provides a window of opportunity to screen these susceptible women and reduce the burden of disease. Screening women during pregnancy is not practiced widely due to concerns of pregnancy loss, bleeding and a lack of clear information among patients as well as healthcare professionals.

## Introduction

Worldwide cervical cancer is the fourth most common cancer among females and there were 604,127 cases in 2020. It is also a leading cause of deaths due to cancer and there were 341,831 deaths in the same year [1]. In a low/low-middle income country (LMIC), pregnancy remains the first point of contact to healthcare for a majority of women. Furthermore, the age specific incidence rate is higher in the age group of 30–39 (reproductive age group). Studies have reported upto 5% incidence of abnormal cervical cytology during pregnancy [2]. The incidence of cervical cancer during pregnancy varies from 3.3 to 26 per 100,000 births. Approximately 1–35 women diagnosed with cervical cancer are pregnant or postpartum and amongst them half are diagnosed antenatally in early stages [3]. In countries with a well-established and effective cervical cancer screening programme, e.g., the National Health Service Cervical Screening Program in the United Kingdom; Pap test is done only for women who missed their previous screening appointments due to difficulties in sampling and interpretation of smear. In United Kingdom, the uptake of cervical cancer screening is around 80% [4]. However, most LMICs are still lacking an effective national screening programme for cervical cancer despite facing a major burden of the disease. This along with a lack of awareness among the population results in screening being largely opportunistic. With a rise in the institutional delivery rates in these nations, pregnancy can be utilised as an opportunity to screen these women.

## Natural Course of Cervical Intraepithelial Neoplasia in Pregnancy

Studies on the natural course of cervical intraepithelial neoplasia (CIN) during pregnancy report a high regression rate (45%–70%) with 5%–15% progressing to CIN2-3 and no or very few cases progressing to invasive cancer. The regression rates were significantly lower in non-pregnant women [5–7]. Diet and nutrition are modifiable risk factors for several cancers and the regression rate of CIN is demonstrated to be influenced by several variables, in particular calcium; zinc; iron; selenium; carotenoids; and vitamins A, B12, C, D, E, and K. Different oligo-elements and micronutrients have demonstrated a potential protective role against cervical cancer by intervening in different stages of the natural history of CIN [8].

### Screening Tools and Challenges

Several authors have used Pap smear as a screening tool. In a prospective study by Priya S et al., Ayre’s spatula was used to collect smear in 200 women. One fourth of the smears were inflammatory and one fifth were unsatisfactory with atypical squamous cells of undetermined significance (ASCUS) and low grade squamous intraepithelial lesion (LSIL) reported in 0.5% each [9]. In a study conducted at our center, authors assessed the acceptability and feasibility of opportunistic cervical cancer screening in pregnancy by conventional Pap and HPV DNA (HC 2) testing. Ayres spatula with endocervical brush was used for Pap smear and cyto-broom for HPV DNA sampling. Abnormal Pap smear was found in 1.5% (4/269) subjects whereas the prevalence of HPV infection was 8.2%. Only one subject had both abnormal cytology and HPV positive result. Colposcopy was performed in all screen positives and only 3 out of 15 cases had a swede score >2. Most women do not undergo screening during pregnancy due to a lack of clear information compounded by fear of bleeding or abortion. Authors faced difficulties in convincing women for testing in 17% and 60% women had apprehension about pain. There were difficulties in visualization of cervix in 31% cases which increased with advancing gestation and excessive discharge was problematic in 14% cases. Pain and discomfort were less at earlier gestational ages ((8% in <28 weeks vs. 38.24% in rest) [10]. In another large retrospective study on 2641 women who underwent a cytology screening during pregnancy, 79 (3.0%) had abnormal results. Of these 70 women, 42 had grade ≥1 CIN. Ayres spatula was the commonest sampling tool followed by cotton swabs [11].

Of the sampling tools, cyto-broom is favoured whereas endocervical brush is not favoured by many due to the associated risk of bleeding which may cause distress. In a randomized controlled trial of 352 pregnant women assigned to cotton swab and Ayres spatula, cytobrush and Ayers spatula, or Cervex-brush; the performance pf Pap smear was better with endocervical brush however with a small increase in the incidence of spotting without any serious adverse events [12].

Cervical hyperaemia and frequent inflammation creates challenges in performing Pap test and smears are difficult to analyse as decidual cells are mistaken for atypia. Arias Stella reaction of pregnancy leads to nuclear changes in endocervical glands of pregnant women (9%–37.5%) which it is characterised by cellular enlargement, pleomorphic cells with large, hyperchromatic nuclei and prominent nucleoli [13].

The early second trimester is thus an ideal time as endocervical cells are translocated outside the cervix, the transformation zone is visualized easily, and sampling is easier at this time. The endocervical sampler used in conventional Pap smear extends only till the lower half of the cervix and various studies have shown its safety during pregnancy [12, 14].

### Role of HPV DNA Testing and Self-Collection

HPV DNA test is widely used for screening in the general population and has recently been endorsed by the World Health Organization as the primary screening modality but its integration during pregnancy may differ due to limited available literature regarding its utility. It has an additional advantage that vaginal self-sampling can be performed which has shown to have good concordance with cervical samples [15, 16]. A major concern with HPV testing in women under 30 years of age has been the higher incidence and prevalence of HPV infection in the third decade of life in most reports worldwide which raises concerns about high rates of triage and unnecessary treatments [17]. Hence Pap smear results are more representative to decide the clinical management of pregnant women while the value of a positive HPV DNA test should be restricted to compose an indication for closer follow-up. A recent literature review of 10 studies also suggested that Pap smear should still be the first-line diagnostic tool during pregnancy [18].

### Role of Colposcopy and Biopsy

Colposcopy is safe to perform but challenging and only an experienced colposcopist should perform as lack of experience could potentially lead to an overestimation. Risk-based threshold for entry to colposcopy are the same, regardless of pregnancy. During pregnancy the squamo-columnar junction and transformation zone are more exposed but visualization of all four quadrants is hindered by oedema, cyanosis, vaginal wall protrusion and mucus. Aim is to exclude invasive cancer. The decision to proceed with a biopsy during pregnancy should be a shared decision with the individual and the colposcopist. There is evidence of safety of cervical biopsy but with a slight risk of excess bleeding and it is indicated when there is a concern for high grade lesion or cancer. Endocervical curettage and endometrial biopsy is not recommended [19–22].

A greater concern may be compliance with and timing of colposcopy. Results from the study by Sudhakaran S et al. suggested that only 68% of women complied with the invitation for colposcopy [10].

## Discussion

Cervical cancer is recognized as a major public health problem and yet systematic screening in LMICs has remained an elusive target. Screening these women even with one or two rounds of testing is a mammoth task, with health facilities that are already stressed with the burden of other communicable and non-communicable diseases.

### Recommendations and Algorithm After a Positive Screen

Routine antenatal and postpartum care should include a review of the woman’s cervical screening history and women who are due or overdue for screening should be screened. Screening can be done at any time during pregnancy, provided that the correct sampling equipment is used. The American and Australian Guidelines suggest that women who are high risk HPV (16/18) positive in pregnancy should be referred for early colposcopy. Others can be advised a cytology triage, which if suggestive of high grade squamous intraepithelial lesion (HSIL)/glandular abnormality can be referred for early colposcopy. Those with a colposcopic impression of CIN 2/3 can be reviewed again in postpartum period or can undergo a surveillance colposcopy every 12–24 weeks [19, 23].

The most recent Canadian Guidelines suggest that pregnant patients who are high risk HPV positive with reflex normal or low-grade cytology (ASCUS or LSIL) should have HPV based screening repeated 3 months post-partum. Pregnant women who are high risk HPV positive with reflex high-grade or glandular cytology (ASC-H, HSIL, AGC) should be seen in colposcopy within 4 weeks [20].

### Referral, Follow Up and Timing of Treatment

Pregnant women should be referred urgently and seen within 2 weeks by a gynaecological oncologist if they have a cytology or colposcopic prediction of invasive disease or a histologically confirmed diagnosis of invasive carcinoma [19, 23]. The role of a gynaecologic oncologist becomes important in such cases to plan a management. Definitive treatment of a suspected high-grade lesion, except invasive cancer, may be safely deferred until after the pregnancy. Optimal timing of treatment in such cases should not be less than four to 6 weeks and preferably at 3 months. In a systematic review and meta-analysis of 20 studies reporting on pregnancy outcome of 12,159,293 women, authors reported that women treated for CIN before or during pregnancy, had a significantly higher risk of preterm birth (OR 1.7, 95% CI 1.0–2.7) and this risk was higher in those treated during pregnancy (OR 6.5, 95% CI 1.1–37) [24].

A recent meta-analysis of eight retrospective studies comprising 813 patients whose premalignant lesions were evaluated cytologically, of whom 685 delivered via the vaginal route, and 233 patients whose squamous intraepithelial lesions were evaluated histologically, of whom 162 delivered vaginally. Regression rates were comparable among women that delivered with caesarean section compared to patients that delivered vaginally, both in the cytological (OR 1.32, 95% CI 0.56, 3.12) and in the histological evaluation (OR 1.87, 95% CI 0.50, 6.96) of the lesions. Subgroup analysis revealed consistent results for all subgroups of premalignant lesions. Finally, the results observed for both the persistence and the progression rates of these lesions were proportional. To conclude, the delivery mode did not alter the natural evolution of squamous intraepithelial lesions in and therefore their presence should not determine the mode of delivery [25].

## Conclusion

Screening for cervical cancer is safe during pregnancy and correct sampling tool must be used to minimize bleeding risk. Colposcopy should be performed by an experienced personnel and biopsy should be done only if there is suspicion of invasive carcinoma. Treatment for suspected/histologic HSIL is not recommended during pregnancy.
